# Exogenous Glycinebetaine Reduces Cadmium Uptake and Mitigates Cadmium Toxicity in Two Tobacco Genotypes Differing in Cadmium Tolerance

**DOI:** 10.3390/ijms20071612

**Published:** 2019-03-31

**Authors:** Xiaoyan He, Marvin E.A. Richmond, Darron V. Williams, Weite Zheng, Feibo Wu

**Affiliations:** 1 Department of Agronomy, College of Agriculture and Biotechnology, Zijingang Campus, Zhejiang University, Hangzhou 310058, China; hexiaoyan@zju.edu.cn (X.H.); m.richmond25@hotmail.com (M.E.R.); darronw3@gmail.com (D.V.W.); 21116045@zju.edu.cn (W.Z.); 2 College of Agronomy, Qingdao Agricultural University, Qingdao 266109, China

**Keywords:** cadmium, glycinebetaine, photosynthesis, ultrastructure, tobacco (*Nicotiana tabacum* L.)

## Abstract

Greenhouse hydroponic experiments were conducted using Cd-sensitive (*cv.* Guiyan1) and Cd-tolerant (*cv.* Yunyan2) tobacco cultivars to study the ameliorative effects of exogenous glycinebetaine (GB) upon 5 μM Cd stress. The foliar spray of GB markedly reduced Cd concentrations in plants and alleviated Cd-induced soil plant analysis development (SPAD) value, plant height and root length inhibition, with the mitigation effect being more obvious in Yunyan2. External GB markedly reduced Cd-induced malondialdehyde (MDA) accumulation, induced stomatal closure, ameliorated Cd-induced damages on leaf/root ultrastructure, and increased the chlorophyll content and fluorescence parameters of Fo, Fm, and Fv/Fm in both cultivars and Pn in Yunyan2. Exogenous GB counteracted Cd-induced alterations of certain antioxidant enzymes and nutrients uptake, e.g., the depressed Cd-induced increase of superoxide dismutase (SOD) and peroxidase (POD) activities, but significantly elevated the depressed catalase (CAT) and ascorbate peroxidase (APX) activities. The results indicate that alleviated Cd toxicity by GB application is related to the reduced Cd uptake and MDA accumulation, balanced nutrients and antioxidant enzyme activities, improved PSII, and integrated ultrastructure in tobacco plants.

## 1. Introduction

A high cadmium (Cd) content in soil results in the inhibition of plant growth and yield reduction [1,2]. Tobacco (*Nicotiana tabacum* L.) is one of the most economically important crops worldwide. However, it is more acclimated to Cd uptake than other crops and preferentially enriches Cd in leaves, readily causing a risk for human health through the inhalation of smoke from cigarettes [3]. It has been demonstrated that Cd-exposed populations through smoking has a 2–3 folds higher morbidity risk of peripheral arterial disease than those of nonsmokers [4]. Accordingly, smoking has become one of the most important absorptive pathways of Cd in humans [5]. Thus, there is an urgent need to develop reliable approaches to prevent Cd accumulation in tobacco. The application of chemical regulators to alleviate Cd toxicity and to reduce plant Cd uptake in medium or slightly contaminated farmlands might offer a cost-effective and practically acceptable strategy for the complete utilization of natural resources and safe tobacco production.

Cd interferes the biochemical and physiological processes, such as mineral uptake [6], photosynthesis [7], oxidative stress [8], stomatal conductance, and transpiration [9]. Cd injury is probably attributed to an alteration in the oxidant level in plants, as Cd may cause reactive oxygen species (ROS), leading to oxidative injury [10]. Correspondingly, the plant internal metabolites and scavenging enzymes of active oxygen are relatively changed, which is beneficial to the development of a defense system. The different influence patterns of Cd toxicity on ROS-scavenging enzymes activities, such as superoxide dismutase (SOD), peroxidase (POD), catalase (CAT), and ascorbate peroxidase (APX), were found [11,12,13]. Therefore, in order to verify the hypothesis that some antioxidants may also be sensitive targets of Cd toxicity besides their function in the detoxification in plants, the changes in Cd-induced oxidant stress and antioxidant systems is imperative to determine.

**Figure 1:**
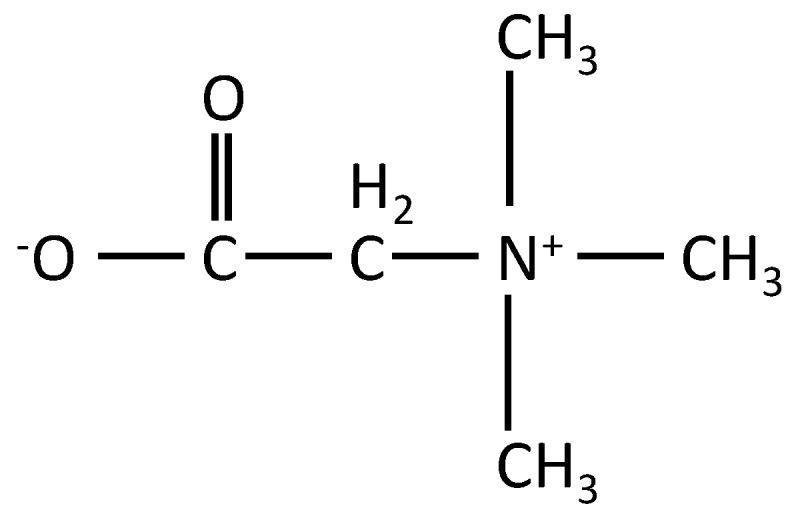
The chemical structure of glycinebetaine.

Glycinebetaine (GB) (Figure 1), being a nitrogenous compound (quaternary amine) that behaves as zwitterion, is known to perform two main functions: osmotic adjustment and cellular compatibility in plants. The natural accumulation of GB in plants is correlated with abiotic stress tolerance [14,15]. A genetic transformation with GB synthesizing enzyme gene(s) in naturally non-accumulating plants has resulted in an enhanced tolerance against a variety of abiotic stresses [16,17]. The application of GB, foliar spray or genetic modification, improves stress tolerance in different plant species and enhances antioxidant defense systems in plant responses to various oxidative stresses [18,19]. Banu et al. [20] found that GB induces the expression of ROS scavenging antioxidant defense genes and suppresses ROS accumulation and cell death in cultured tobacco cells exposed to NaCl stress. As to Cd stress, Duman et al. [21] observed that exogenous GB and trehalose reduced the deleterious effects of Cd stress in duckweed (*Lemna gibba*). Islam and colleagues [22,23] reported that exogenous proline and glycinebetaine increase ascorbate-glutathione cycle and antioxidant enzyme activities and confer tolerance to Cd stress in cultured tobacco cells. However, the mechanism by which GB confers tolerance to plants against heavy metal stress, including Cd stress, is still poorly understood. Therefore, the question arises whether GB participates in Cd tolerance and whether GB application could reduce Cd accumulation in tobacco plants. It is also of great significance to understand whether exogenous GB can be used as a regulator of Cd stress or as an antioxidant intervention strategy in preventing oxidative stress in responses to Cd stress so as to better understand how plants adapt to adverse environments.

The present study was conducted via a hydroponic experiment to investigate the potential role of exogenous GB in alleviating Cd-induced changes in antioxidative metabolism, ultrastructure, photosynthesis, and chlorophyll fluorescence of two tobacco cultivars of Cd-sensitive (*cv.* Guiyan1) and Cd-tolerant (*cv.* Yunyan2). We aimed to provide a basis for developing strategies to reduce the risks associated with Cd toxicity and maintaining a sustainable tobacco production.

## 2. Results

### 2.1. Effect of Exogenous GB on Cd-Induced Suppression in Plant Growth

**Figure 2:**
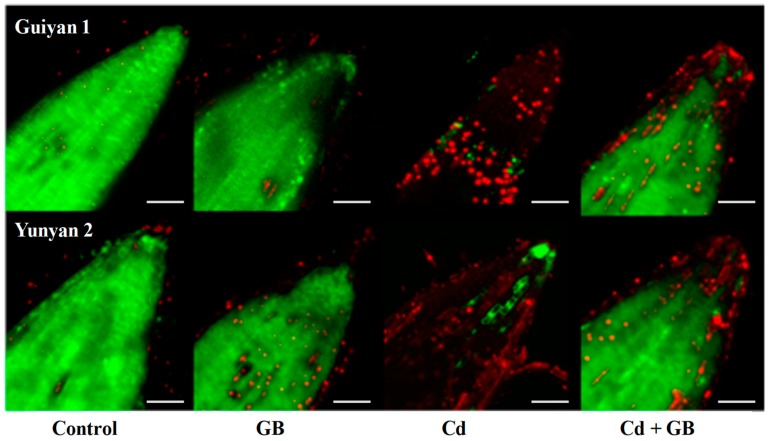
The effects of Cd and exogenous GB on the cell vigor in the root tips of Guiyan1 and Yunyan2 detected by an flurescein diacetate-propidium iodide (FDA-PI) dual fluorescent dye after 15 days of Cd exposure: The green and red fluorescence indicate the fluorescent dye in living and dead cells, respectively. Control, GB, Cd, and Cd + GB correspond to BNS + foliar spray of deionized water, BNS + foliar spray of 500 μM GB, BNS + foliar spray of deionized water + 5 μM CdCl_2_, and BNS + foliar spray of 500 μM GB + 5 μM CdCl_2_, respectively. Scale bars = 250 µm. The figure is representative of three different experiments.

After 15 days of Cd exposure, Cd toxicity markedly hindered the soil plant analysis development (SPAD) value, plant height, root length, and biomass (Table 1). The tolerant cultivar Yunyan2 was less affected in terms of the abovementioned growth traits, whereas the sensitive Guiyan1 was more affected. Cd + GB treatment apparently alleviated the Cd-induced SPAD value, plant height, and root length inhibition. The alleviating effects were evaluated using the formula-based integrated relation. There is a positive correlation between the alleviating effects and the integrated scores. According to the integrated scores, Yunyan2 had a better mitigation effect under Cd + GB with a higher score than Guiyan1 (Table 1). A parallel experiment was performed to evaluate the vigor of root cells using flurescein diacetate-propidium iodide (FDA-PI) assay (Figure 2). After 15 days of Cd treatment, bright and red fluorescence were observed in roots of tobacco seedlings grown in Cd media, and Yunyan2 showed relatively few red fluorescence compared with Guiyan1. Cd + GB treatment markedly decreased the red fluorescence intensity but increased the green fluorescence compared with Cd alone treatment. The red fluorescence was very low, but detectable levels of red fluorescence were observed in the Cd + GB roots.

### 2.2. Effect of Exogenous GB on Cd and Nutrient Elements Contents in Tobacco Seedlings

The 2 tobacco cultivars had clear differences in Cd concentration: Yunyan2 recorded 2%, 21%, and 8% more than Guiyan1 in S1 (three top leaves with stem), S2 (middle part of shoot), and S3 (three bottom leaves with stem), but Guiyan1 recorded 85%, 23%, and 1% more than Yunyan2 in R1 (three cm of root tip), R2 (middle part of root), and R3 (three cm of root bottom) (Table 2). Exogenous GB markedly suppressed Cd concentration compared with the Cd alone treatment, i.e., in S1, S2, and S3, the reductions were 14%, 20%, and 17% in Guiyan1 and 9%, 21%, and 7% in Yunyan2, and in R1, R2, and R3, these were 15%, 7%, and 8% and 15%, 13%, and 7%, respectively (Table 2). Cd treatment markedly decreased the shoot and root Zn and Mn in Guiyan1 and Yunyan2 and decreased the root Cu in Guiyan1 and shoot Cu in Yunyan2 but increased the shoot Fe and Cu and root Fe in Guiyan1 and root Fe and Cu in Yunyan2. However, to a degree, exogenous GB application recovered the shoot/root Fe and Cu in Guiyan1 and the shoot Zn and root Fe in Yunyan2 (Table 2).

### 2.3. Effect of Cd and Exogenous GB on MDA Accumulation and Certain Antioxidant Enzyme Activities

Cd treatment displayed a significant accumulation of MDA in tobacco seedlings, which was decreased with the application of exogenous GB (Supplemental Figure S1). Averaged over the 3 sampling dates (5, 10, and 15 days of Cd exposure), the shoot/root MDA contents in Cd + GB were significantly decreased by 8%/6% and 15%/6% in Guiyan1 and Yunyan2, respectively, compared with Cd alone treatment.

**Figure 3:**
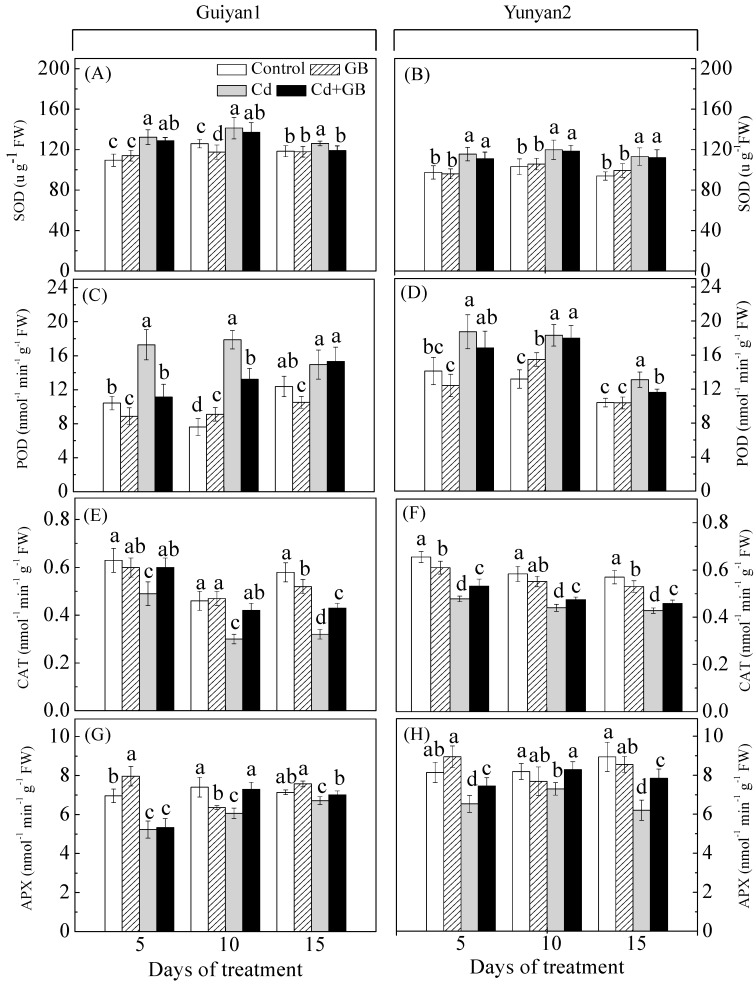
The effects of Cd and exogenous GB application on the superoxide dismutase (SOD) (**A**,**B**), peroxidase (POD) (**C**,**D**), catalase (CAT) (**E**,**F**), and ascorbate peroxidase (APX) (**G**,**H**) activity in the roots of two tobacco cultivars exposed to 5 µM Cd for 5, 10, and 15 days: The error bars represent the SD values (*n* = 3). The different letters indicate the significant differences (*p* < 0.05) among the 4 treatments within each sampling date. Control, GB, Cd, and Cd + GB correspond to BNS + foliar spray of deionized water, BNS + foliar spray of 500 μM GB, BNS + foliar spray of deionized water + 5 μM CdCl_2_, and BNS + foliar spray of 500 μM GB + 5 μM CdCl_2_, respectively.

Cd stress significantly promoted shoot and root SOD and POD activities in Guiyan1 and Yunyan2. Cd + GB treatment decreased the SOD and POD activities at different levels (Figure 3; Supplemental Figure S2). For example, the root POD activities were significantly decreased by 36% and 26% in Guiyan1 after 5 and 10 day of GB + Cd treatment, respectively, while were decreased by 11% in Yunyan2 after 15 day of GB + Cd treatment compared with Cd alone treatment (Figure 3). Averaged over the 3 sampling dates, the CAT/APX activities of Guiyan1 and Yunyan2 under Cd alone treatment were 16%/14% and 19%/12% in the shoots and 29%/16% and 25%/14% in the roots, respectively, lower than the controls. GB addition improved the CAT and APX activities compared with Cd alone treatment. Averaged over the 3 sampling dates, the CAT activity in the shoots/roots increased by 7%/5% and 13%/9% in Guiyan1 and Yunyan2 under Cd + GB, respectively, and some of the CAT activities in Cd + GB were even closed to the control and GB alone groups (Figure 3; Supplemental Figure S2).

### 2.4. Effect of Cd and Exogenous GB on Chlorophyll Fluorescence and Photosynthetic Parameters

**Figure 4:**
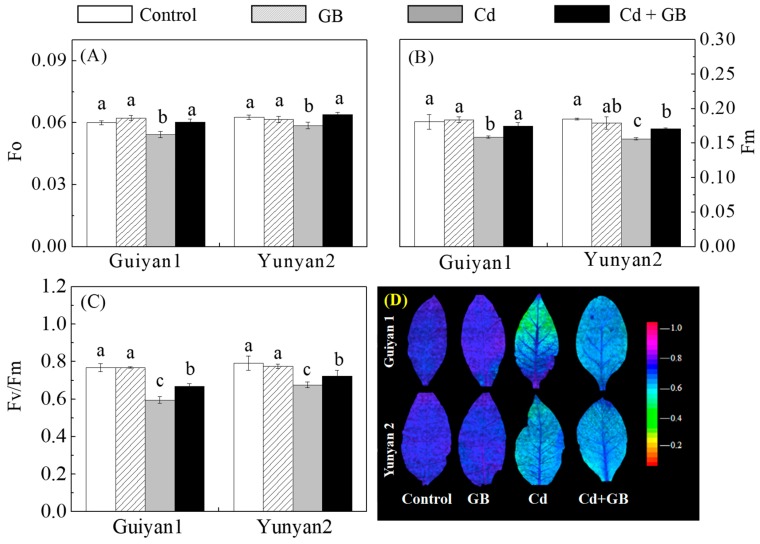
The effects of Cd and exogenous GB on the fluorescence parameters (**A**–**C**) and the Fv/Fm false color images (**D**) of Guiyan1 and Yunyan2 after 15 days of Cd exposure. Fo, minimal fluorescence yield (**A**); Fm, maximal fluorescence yield (**B**); and Fv/Fm, maximum quantum yield of PSII (**C**). The error bars represent the SD values (*n* = 3). The different letters indicate the significant differences (*p* < 0.05) among the 4 treatments within each cultivar. Control, GB, Cd, and Cd + GB correspond to BNS + foliar spray of deionized water, BNS + foliar spray of 500 μM GB, BNS + foliar spray of deionized water + 5 μM CdCl_2_ and BNS + foliar spray of 500 μM GB + 5 μM CdCl_2_, respectively. The figure is representative of three different experiments.

Cd markedly reduced Pn, gs, Ci, and Tr by 23%, 34%, 9%, and 36% in Guiyan1 and 16%, 48%, 12%, and 30% in Yunyan2, respectively, compared with the controls. GB (Cd + GB) increased Pn and Ci of Yunyan2 by 9% each compared with Cd alone treatment (Supplemental Figure S3). Meanwhile, compared with the controls, Cd significantly decreased Fo, Fm, and Fv/Fm in Guiyan1 and Yunyan2, whereas, GB application improved Fo, Fm, and Fv/Fm by 11%, 10%, and 17% and by 8%, 10%, and 6% in Guiyan1 and Yunyan2, respectively, compared with the Cd alone treatment (Figure 4). False color images visually depicted the changes in Fv/Fm. The leaf color changed from blue to green with a decrease in the Fv/Fm ratio under Cd stress, especially for Guiyan1, but it was recovered obviously after GB application (Figure 4).

### 2.5. Effect of Cd and Exogenous GB on the Ultrastructure of Roots and Leaves

**Figure 5:**
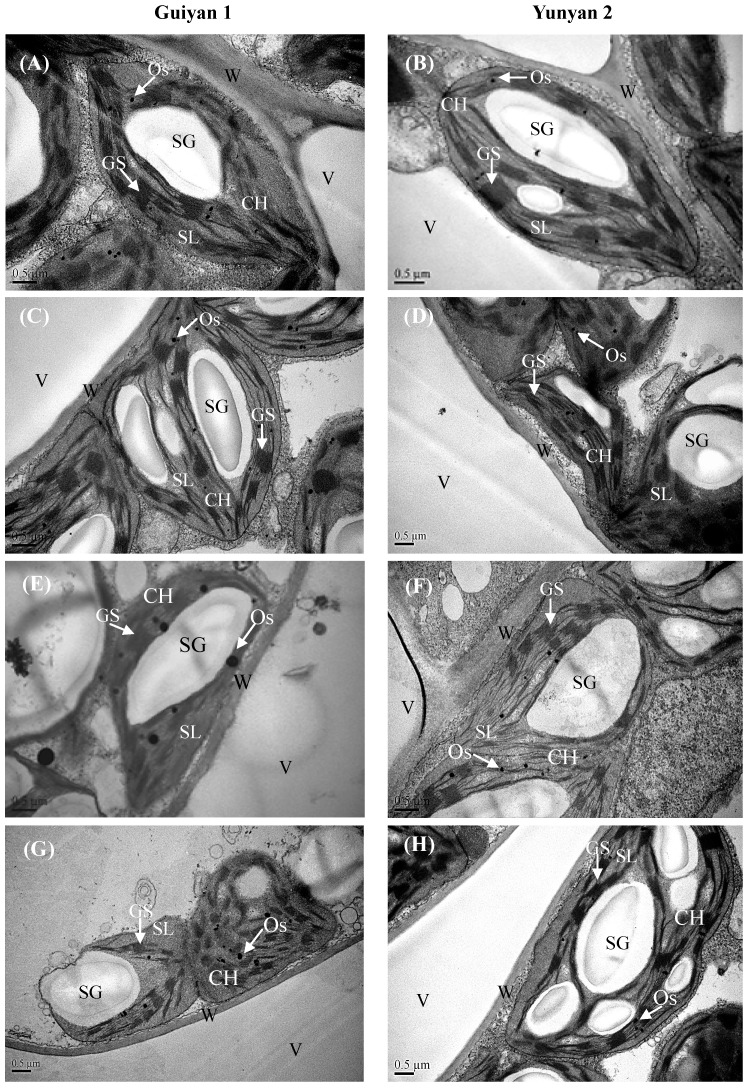
Transmission electron micrograph images of the leaf mesophyll cells of Guiyan1 (left panel) and Yunyan2 (right panel) after 15 days of Control (**A**,**B**), GB (**C**,**D**), Cd (**E**,**F**), and Cd + GB (**G**,**H**) treatments, respectively. Control, GB, Cd and Cd + GB correspond to BNS + foliar spray of deionized water, BNS + foliar spray of 500 μM GB, BNS + foliar spray of deionized water + 5 μM CdCl_2_, and BNS + foliar spray of 500 μM GB + 5 μM CdCl_2_, respectively. Labels: CH, chloroplast; GS, grana stack; SL, stroma lamella; Os, osmiophilic plastolobuli; SG, starch grain; V, vacuole; W, cell wall. Bar = 0.5 μm.

In control and GB-alone conditions, there were oval chloroplasts with a regular arrangement of thylakoid membranes of the stroma and of the grana in the spongy mesophyll cells and large starch grains and few osmiophilic plastoglobuli in the chloroplasts. After 15 days of Cd exposure, an irregular outline of chloroplasts could be found, and thylakoid membranes were dissolved and rarely visible; the cell wall was also distorted, and the osmiophilic plastoglobulis were much bigger than controls, especially in Guiyan1. The foliar application of GB obviously alleviated Cd-induced chloroplast damage in Yunyan2 but had little effect on Guiyan1 (Figure 5). After applying Cd to basic nutrition solution (BNS), some changes were also noted in the root cells (Supplemental Figure S4). An irregular outline of the cell structure and nuclear membrane was observed, nucleoli and karyoplasms were loosened, plasmolysis was evident, and a number of vacuoles accumulated along with the emergence of many great electron dense granules (EDG). Adding GB showed that cell structure and nuclei were better formed than those of the Cd alone treatment in Yunyan2. However, exogenous GB had a very little mitigation on the Cd-induced root cell changes in Guiyan1 (Supplemental Figure S4). Observations of the leaf surface of two tobacco cultivars by SEM revealed that stomatal movement could be regulated by Cd and exogenous GB application in Guiyan1 and Yunyan2 (Supplemental Figure S5). In the control condition, most of stomata were open; however, almost all the stomata were closed under Cd + GB treatment.

## 3. Discussion

### 3.1. Exogenous GB Reduces Cd Concentration in Tobacco Plants and Alleviates Cd-Induced Suppression in Plant Growth and Counteracts Nutrient Elements Changes

With tobacco being one of the susceptible plants to Cd stress, strategies for reducing Cd accumulation and the related health risks are urgently desired [3,4]. GB is water-soluble, nontoxic, and environmentally safe. It can easily be procured as a relatively inexpensive by-product from sugar beets. A number of reports have demonstrated that the exogenous application of GB improves stress tolerance [22,23]. In this study, Cd concentrations in the shoots and roots of both tobacco cultivars were reduced by GB application, and the mitigation effect was more obvious in Yunyan2. These results indicate the pronounced role of exogenous GB in protecting Cd toxicity and its potential use for reducing Cd concentrations in tobacco plants (Figure 2, Table 2). Many studies on the interaction between the uptake and distribution of Cd and essential mineral elements in crops have been reported, but the results are inconsistent. Previous studies have shown that Zn, Mn, and Cu interfere with the uptake of Cd and the translocation from roots to shoots [24,25]. Similar to our current study, Cd application significantly decreased leaf/root Zn, Mn, and Cu contents but increased Fe content, which led to an imbalance of the mineral content in plants through a disturbed metal uptake and allocation (Table 2). Exogenous GB application, to a degree, recovered shoot/root Fe, Cu, and Zn contents caused by Cd stress, indicating that GB can mitigate Cd toxicity partly through balancing the metabolism of elements.

### 3.2. Exogenous GB Counteracts Cd-Induced Alterations in the Antioxidant System

Oxidative stress is a central part of abiotic and biotic stresses. This mechanism is caused by a serious cell imbalance between the production of ROS and antioxidative enzymes, which leads to dramatic physiological disorders. In our study, the inhibition in plant growth by Cd was linked with Cd-induced MDA accumulation (Supplemental Figure S1), which indicates that Cd may cause oxidative damage to plants. However, the exogenous application of GB resulted in a significant reduction in MDA levels, and a higher reduction ratio was detected in Yunyan2 shoot. Results of cell viability in roots were consistent with the measurements on Cd stress-generated MDA. This result indicated that GB has a potential mitigation role on Cd-induced oxidative stress through decreasing the MDA content in tobacco seedlings.

The enzymatic ROS scavenging system plays an important role in maintaining the structure and function of membrane and cellular redox equilibrium [26]. In the present study, Cd treatment promoted SOD and POD activities but suppressed CAT and APX activities in the shoots and roots of both tobacco cultivars; however, the pattern of alterations in their activities induced by Cd stress was counteracted in the presence of GB (Figure 3; Supplemental Figure S2). Previous investigations observed that the application of GB could alleviate the oxidative stress induced by heavy metals [27,28]. The high SOD and POD decreasing ratio in Guiyan1 roots could be attributable to the excessive production of ROS, resulting in a greater growth inhibition under Cd + GB. However, Yunyan2 showed a relatively better ROS scavenging capacity under Cd stress than Guiyan1 because of its higher promoted activities of CAT under Cd + GB and lower decrease of APX activities under the Cd alone treatment. All these results indicated that the greater tolerance of Yunyan2 to Cd might be brought about by its strong antioxidant enzyme system compared with that of Guiyan1 and that GB can harmonize the activities of antioxidant enzymes and protect cells and tissues against oxidative damage caused by oxidative stress.

### 3.3. Exogenous GB Offsets Cd-Induced Inhibition in Photosynthesis and ChlorophyII Fluorescence Characteristics

Photosynthetic and chlorophyll fluorescence parameters are considered powerful tools for studying the physiological responses of plants in response to metal stress and for providing a direct method for evaluating photosynthetic activities [29,30]. In this study, Cd stress negatively influenced various photosynthetic parameters like Pn, Ci, and Tr (Supplemental Figure S3), and the application of GB alleviated the Cd-induced decrease of Pn in Yunyan2. However, Guiyan1 had higher gs and Tr than Yunyan2 under Cd + GB, suggesting that a Cd-induced photosynthetic system impairment was rehabilitated by exogenous GB fortification, possibly through maintaining the photosynthetic capacity by regulating stomatal conductance and decreasing the transpiration rate, which in turn countered the uptake of Cd.

Photosystem II (PSII) is believed to play a key role in the response of leaf photosynthesis to environmental perturbations [31]. Reducing PSII is considered the main target of Cd toxicity stress in plants [17]. Similar results were found in the present study, i.e., Cd significantly reduced Fo, Fm, and Fv/Fm in 2 tobacco cultivars (Figure 4). Reduced values of Fv/Fm also indicate that Cd-induced stress impaired the maximum quantum efficiency of PSII. A greater decrease of Fo and Fv/Fm in Guiyan1 demonstrated that the PSII of Guiyan1 was more sensitive to Cd stress, suggesting that the high tolerance of Yunyan2 was partly attributable to the higher protective capacity of PSII. Wang et al. [32] found that the enhanced drought tolerance was due to the accelerated recovery of the PSII from a photoinactivated state by GB. In this study, the foliar application of GB significantly alleviated the alteration of PSII caused by Cd stress. The results provided evidence that the application of GB can protect of PSII, contributing to Cd tolerance in tobacco plants.

### 3.4. Exogenous GB Mitigates Cd-Induced Damage in Cell Ultrastructure

The structure of the chloroplast has a relationship with the photochemistry activity. Thylakoid membrane leakage under Cd stress might be responsible for the reduced photosynthetic parameters, and it is the first limiting factor for photosynthesis [33]. In our current study, abnormal-shaped chloroplasts, dissolved thylakoid membranes, and bigger osmiophilic plastolobuli were observed under Cd stress, especially in Guiyan1, and the foliar spray of GB alleviated these damages particularly in Yunyan2 (Figure 5). This explains the higher photosynthetic activity in Yunyan2 under the Cd alone and Cd + GB treatments than Guiyan1. On the other hand, the stomatal movement is traditionally considered to be tightly regulated and to reflect the level of abiotic stress [34]. Although both the cultivars did not exhibit significant differences in stomatal movements (Supplemental Figure S5), the application of GB led to the osmotically driven changes in cell turgor which mediates stomatal movements with closed stomata, leading to a decreased stomatal conductance and resulting in an improved plant growth under Cd stress.

The nucleus is the genetic center for all eukaryotes. Experiencing the toxicity of Cd, the damage to the root nucleus was serious in 2 tobacco cultivars, reflected in the irregular cell structure, loose nucleoli and karyoplasms, large vacuoles, great electron dense granules, more mitochondrion, and plasmolysis (Supplemental Figure S4). The application of GB to Yunyan2 alleviated most of the disorder caused by Cd stress, but exogenous GB had very little effect on mitigating Guiyan1 disorders, as even bigger and larger vacuoles were formed under Cd + GB condition. The vacuoles of plants are considered to be the organelles in which the nourishment is accumulated and stored. However, when heavy metals such as Cd exist in the cell, the superfluous Cd in the cytoplasm could be stockpiled in the vacuoles [35]. The large and well-shaped vacuoles indicate vacuole compartmentation might be a probable mechanism of Cd detoxification in Guiyan1, explaining the high Cd content in its roots.

## 4. Materials and Methods

### 4.1. Plant Material and Experimental Designs

The greenhouse hydroponic experiment was carried out on Zijingang Campus, Zhejiang University, Hangzhou, China. Healthy tobacco seeds of Guiyan1 (Cd-sensitive) and Yunyan2 (Cd-tolerant) (Lab of Prof. Guoping Zhang, Department of Agronomy of Zhejiang University, Hangzhou China) were germinated in sterilized, moist vermiculite in a growth chamber at 25 °C/20 °C (day/night). Uniform healthy 4-leaf stage (50 day old) seedlings were transplanted to 5-L containers filled with 4.5-L basal nutrient solution (BNS), and the containers were placed in a greenhouse. The composition of BNS was the same as in the study by Liu et al. [25]. The solution pH was adjusted to 5.8 ± 0.1 with HCl or NaOH as required. After 15 days of transplanting, 4 treatments were performed: control (BNS+ foliar spray of deionized water), GB (BNS + foliar spray of 500 μM GB), Cd (BNS + foliar spray of deionized water + 5 μM CdCl_2_), and Cd + GB (BNS + foliar spray of 500 μM GB + 5 μM CdCl_2_). The foliar spray of deionized water and GB were conducted one day before Cd treatment and 1 and 3 days after Cd treatment. The experiment was laid in a split-plot design with treatment as the main plot and genotype as the subplot with three replicates for each treatment. The nutrient solution was continuously aerated with pumps and renewed every 5 d. After 5, 10, and 15 days of treatment, the fresh plant samples were immediately frozen in liquid nitrogen and stored frozen at −80 °C for the determination of antioxidative enzyme activities and MDA contents. Meanwhile, the growth and photosynthesis parameters were measured for the plants after 15 d treatment, and leaf/root ultrastructure were also performed.

### 4.2. Chlorophyll Content and Growth Measurement and Metal Analysis

After 15 days of treatment, the upper second fully opened leaves were selected to measure the SPAD values (chlorophyll meter readings) with three replicates using a chlorophyll meter Minolta SPAD-502 (Minolta, Tokyo, Japan). After measuring the plant heights and root lengths, the roots were soaked in 20 mM Na_2_-EDTA for 3 h and rinsed thoroughly with deionized water. Then plants were separated into roots and shoots, and the fresh weights were measured simultaneously. The roots and shoots were dried at 80 °C and weighed. The dried roots and shoots were ground and ashed at 550 °C for 8 h and then digested with 30% HNO_3_. The Cd and metal concentrations were determined using flame atomic absorption spectrometry (Shimadzu AA-6300, Shimadzu, Kyoto, Japan).

### 4.3. Assay of MDA Content and Enzyme Activities

To determine the enzyme activity, after 5, 10, and 15 days of treatments, the plant fresh roots and shoots were homogenized in 8 mL of a 50 mM sodium phosphate buffer (PBS, pH 7.8) using a prechilled mortar and pestle and subsequently centrifuged at 10,000× *g* for 20 min at 4 °C. The supernatant was used for the assays of all enzyme activities. The SOD, POD, CAT, and APX activities were determined as described by Zeng [36]. To analyze the MDA content, the thiobarbituric acid reaction was measured, which reflected the level of lipid peroxidation. The plant fresh roots and shoots were homogenized and extracted in 10 mL of 0.25% thiobarbituric acid. The extract was heated at 95 °C for 30 min and then quickly cooled on ice. After centrifugation at 10,000× *g* for 10 min, the absorbance of the supernatant was measured at 532 nm. A correction of the nonspecific turbidity was made by subtracting the absorbance value taken at 600 nm [13].

### 4.4. Chlorophyll Fluorescence and Photosynthetic Parameters

The chlorophyll fluorescence parameters were determined using an IMAGING-PAM chlorophyll fluorometer (Walz, Effeltrich, Germany) [37]. The leaves were kept in the dark for 20 min before measurement. The minimal fluorescence level (Fo) was measured by measuring light (<0.05 μmol m^−2^ s^−1^ PAR), and the maximal fluorescence level (Fm) was determined by a saturating pulse (2500 μmol m^−2^ s^−1^ PAR). The actinic light intensity was set as 280 µmol mol^−2^ s^−1^ PAR. Variable fluorescence was calculated as Fv = Fm − Fo; the maximal quantum yield of PSII photochemistry was Fv/Fm = (Fm − Fo)/Fm. The Fv/Fm false color images were created by ImagingWin software (IMAGING-PAM, Walz, Effeltrich, Germany). The net photosynthetic rate (Pn), transpiration rate (Tr), stomatal conductance (gs), and intracellular CO_2_ concentration (Ci) were measured by a portable photosynthesis system LI-6400 (LI-COR, Lincoln, NE, USA). All the measurements were carried out with the upper second fully expanded leaves after 15 days of Cd treatments.

### 4.5. Determination of Cell Viability

To evaluate cell viability, after 15 days of Cd exposure, the root tips were rinsed 3 times with deionized water and blotted dry gently, then treated by staining with fluorescein diacetate and propidium iodide (FDA-PI) for 40 min, and washed 3 times with deionized water for 5 min. Red and green fluorescence and concurrent differential interference contrast images were obtained with a Zeiss LSM 780 fluorescent microscope (Zeiss, Oberkochen, Germany) with an excitation at 488 nm and an emission at 514 nm. The nonfluorescent esterase substrate FDA was cleaved by esterases in viable cells, releasing fluorescein which stains the cells green, while the characteristics of PI were totally opposite with FDA, which may interact with DNA/RNA in cells, leaving the red fluorescence of dead cells [38].

### 4.6. Ultrastructural Studies Using Electron Microscopy

Small sections (1 mm^2^) from the middle of the upper second fully expanded leaves and root tips (1–3 mm) were used for TEM studies. The samples were fixed in 2.5% glutaraldehyde in a phosphate buffer (pH 7.2) for 6–8 h, postfixed in 1% OsO_4_ (Osmium (VIII) oxide) for 1 h at 4 °C, and then thoroughly washed 3 times with the same buffer. Dehydration was carried out in a graded ethanol series, and then, the samples were infiltrated and embedded in Spurr’s resin. Also ultrathin sections (about 70–90 nm) were cut on an Ultracut E ultramicrotome (Reichert-Jung, Vienna, Austria) and were placed on Formvar-coated copper grids, then were stained with uranyl acetate and lead citrate, and finally examined and photographed with a JEM-1230 (JEOL, Tokyo, Japan) electron microscope.

For the SEM studies, small portions (1 mm^2^) from the middle of the upper second fully expanded leaves were selected and treated in the same way as described above for the TEM analysis. After a graded dehydration, the samples were transferred to the mixture of alcohol and isoamyl acetate (*v/v* = 1/1) for about 30 min, then transferred to pure isoamyl acetate for about 1–2 h, and followed by dehydration in a Hitachi ModelHCP-2 critical point dryer with liquid CO_2_. In the end, the specimen was coated with gold-palladium in a Eiko Model IB5 ion coater for 4–5 min and then observed in a scanning electron microscope TM-1000 (Hitachi, Tokyo, Japan).

### 4.7. Statistic Analysis

The statistical analyses were performed by Data Processing System statistical software package with ANOVA followed by Duncan’s multiple range tests to evaluate the significant treatment effects at a significance level of *p* ≤ 0.05. To evaluate the alleviating effects, the following formula-based integrated relation was used: The absolute values of (SPAD value × 0.2 + shoot height × 0.2 + root length × 0.2 + fresh weight × 0.2 + dry weight × 0.2) was adopted [39].

## 5. Conclusions

In conclusion, exogenous GB application significantly alleviated Cd-induced growth inhibition in 2 tobacco cultivars especially for the tolerant cultivar Yunyan2. The alleviation mechanism of GB to Cd stress is associated with (1) a decreased Cd accumulation and a balanced nutrient status; (2) the amelioration of Cd-induced damages on leaf and root ultrastructure, an improved membrane-stabilizing/integrity, an increased SPAD value, and chlorophyll fluorescence including Fv/Fm, Fo, and Fm in both cultivars and Pn in Yunyan2; (3) the partially reversed Cd-induced changes in antioxidant enzyme activities and depressed MDA content compared with Cd alone treatment. The results suggest a potential role for GB as a potent alleviator in plants in responses to Cd stress. Ultimately, the decrease of Cd by GB is expected to have contributions to the reduction of Cd toxicity to humans.

## Figures and Tables

**Table 1:** The effects of exogenous GB on the growth of Cd-treated tobacco seedlings after 15 days of Cd exposure.

Treatment	SPAD Value		Plant Height (cm)		Root Length (cm)		Fresh Weight (g·plant^−1^)				Dry Weight (g·plant^−1^)				Integrated Score *
							Shoot		Root		Shoot		Root		
**Guiyan1**															
Control	41.46 ± 2.76	a	39.13 ± 0.58	a	37.40 ± 1.35	a	71.34 ± 1.99	a	19.98 ± 0.95	a	7.57 ± 0.61	a	1.15 ± 0.09	a	43.61
GB	42.16 ± 1.84	a	37.03 ± 1.18	b	35.11 ± 1.56	a	69.09 ± 1.62	a	15.84 ± 1.20	b	7.43 ± 0.40	a	1.11 ± 0.10	a	41.55
Cd	34.02 ± 3.72	b	20.66 ± 1.25	d	22.95 ± 1.81	c	39.47 ± 1.08	b	9.86 ± 0.69	c	5.08 ± 0.47	b	0.61 ± 0.11	b	26.53
Cd + GB	39.72 ± 3.80	a	25.03 ± 1.03	c	26.03 ± 0.32	b	39.76 ± 1.43	b	9.91 ± 0.76	c	5.41 ± 0.36	b	0.68 ± 0.01	b	29.31
**Yunyan2**															
Control	45.50 ± 3.41	a	40.30 ± 1.42	a	34.75 ± 1.44	a	65.11 ± 1.68	a	15.57 ± 0.51	a	7.52 ± 0.41	a	1.19 ± 0.07	a	41.99
GB	45.88 ± 2.28	a	38.52 ± 0.86	a	34.07 ± 1.65	a	67.86 ± 2.30	a	15.13 ± 0.77	a	7.43 ± 0.36	a	1.15 ± 0.10	a	42.01
Cd	37.60 ± 2.98	b	28.60 ± 0.77	c	27.69 ± 1.54	b	46.27 ± 2.09	b	10.72 ± 1.56	b	4.78 ± 0.59	b	0.70 ± 0.00	c	31.27
Cd + GB	43.80 ± 2.77	a	31.10 ± 1.00	b	33.51 ± 1.14	a	48.12 ± 0.79	b	11.13 ± 0.65	b	4.95 ± 0.54	b	0.83 ± 0.02	b	34.69

The data were the means of three independent replicates. The different letters in each column indicate the significant differences (*p* < 0.05) among the 4 treatments within each cultivar. Control, GB (glycinebetaine), Cd, and Cd + GB correspond to the basic nutrition solution (BNS) + foliar spray of deionized water, BNS + foliar spray of 500 μM GB, BNS + foliar spray of deionized water + 5 μM CdCl_2_, and BNS + foliar spray of 500 μM GB + 5 μM CdCl_2_, respectively. * Integrated score = the absolute values of (the soil plant analysis development (SPAD) value × 0.2 + shoot height × 0.2 + root length × 0.2 + fresh weight × 0.2 + dry weight × 0.2).

**Table 2:** The effects of Cd and exogenous GB on the element concentrations in the shoots and roots of tobacco seedlings at day 15.

Elements (mg·kg^−1^ DW)		Guiyan1								Yunyan2							
		Control		GB		Cd		Cd + GB		Control		GB		Cd		Cd + GB	
**Shoot**																	
Cd	S1	0.05 ± 0.01	d	0.03 ± 0.01	d	11.13 ± 0.09	a	9.56 ± 0.24	c	0.07 ± 0.00	d	0.08 ± 0.00	d	11.36 ± 0.75	a	10.33 ± 0.46	b
	S2	0.04 ± 0.01	d	0.04 ± 0.01	d	10.65 ± 0.59	b	8.52 ± 0.30	c	0.07 ± 0.01	d	0.06 ± 0.03	d	12.89 ± 0.77	a	10.15 ± 0.26	b
	S3	0.05 ± 0.02	d	0.03 ± 0.02	d	12.93 ± 0.71	b	10.74 ± 0.42	c	0.08 ± 0.01	d	0.06 ± 0.01	d	14.05 ± 0.25	a	13.02 ± 0.36	b
Zn	S1	13.39 ± 0.54	d	12.85 ± 0.68	d	10.43 ± 0.42	e	10.26 ± 0.29	e	22.68 ± 0.52	b	25.79 ± 0.61	a	16.35 ± 0.76	c	15.83 ± 0.76	c
	S2	15.36 ± 0.62	bc	17.73 ± 0.90	a	9.41 ± 0.47	d	9.08 ± 0.76	d	15.96 ± 0.25	b	15.87 ± 0.48	b	14.71 ± 0.15	c	17.82 ± 0.22	a
	S3	26.69 ± 0.64	a	25.70 ± 0.11	b	10.51 ± 0.38	e	9.12 ± 0.13	f	22.49 ± 0.09	c	22.68 ± 0.72	c	16.72 ± 0.33	d	16.15 ± 0.67	d
Mn	S1	12.02 ± 0.31	c	11.10 ± 0.54	d	9.73 ± 0.27	e	9.68 ± 0.40	e	15.68 ± 0.17	a	14.90 ± 0.47	b	7.53 ± 0.60	f	7.42 ± 0.26	f
	S2	12.76 ± 0.56	bc	12.30 ± 0.47	c	10.58 ± 0.63	d	10.53 ± 0.56	d	14.47 ± 0.49	a	13.60 ± 0.77	ab	10.59 ± 0.41	d	10.00 ± 0.20	d
	S3	23.85 ± 0.38	a	22.66 ± 0.91	b	14.09 ± 0.98	e	13.80 ± 0.47	e	20.45 ± 0.57	c	19.21 ± 0.23	d	12.48 ± 0.19	f	10.51 ± 0.72	g
Cu	S1	2.74 ± 0.25	c	2.70 ± 0.26	c	5.60 ± 0.18	a	5.29 ± 0.25	a	5.32 ± 0.47	a	5.52 ± 0.15	a	4.45 ± 0.05	b	4.18 ± 0.18	b
	S2	2.67 ± 0.25	e	3.31 ± 0.22	cd	4.01 ± 0.45	b	2.70 ± 0.24	de	5.24 ± 0.19	a	4.93 ± 0.46	a	4.93 ± 0.30	a	3.70 ± 0.54	bc
	S3	4.32 ± 0.76	bcd	3.84 ± 0.52	cde	5.61 ± 0.32	a	3.08 ± 0.93	e	4.52 ± 0.34	bc	5.03 ± 0.44	ab	3.48 ± 0.45	de	4.42 ± 0.00	bc
Fe	S1	13.54 ± 0.42	b	8.18 ± 0.39	d	10.17 ± 0.94	c	8.97 ± 0.34	d	13.21 ± 0.79	b	13.32 ± 0.74	b	19.28 ± 0.55	a	10.18 ± 0.79	c
	S2	12.16 ± 0.41	cd	10.22 ± 0.81	ef	11.16 ± 1.32	de	8.56 ± 0.48	g	12.66 ± 0.67	c	19.38 ± 0.40	a	17.95 ± 0.71	b	9.86 ± 0.29	f
	S3	11.94 ± 0.73	d	10.65 ± 0.94	e	17.12 ± 0.75	c	8.37 ± 0.67	f	21.10 ± 0.34	b	24.47 ± 0.68	a	20.99 ± 0.30	b	12.10 ± 0.55	d
**Root**																	
Cd	R1	0.07 ± 0.00	e	0.07 ± 0.01	e	68.45 ± 1.76	a	58.38 ± 1.15	b	0.05 ± 0.01	e	0.05 ± 0.01	e	36.99 ± 1.04	c	31.38 ± 0.60	d
	R2	0.06 ± 0.01	e	0.08 ± 0.01	e	62.76 ± 0.22	a	58.11 ± 0.39	b	0.06 ± 0.02	e	0.04 ± 0.01	e	50.59 ± 1.70	c	44.00 ± 2.20	d
	R3	0.05 ± 0.01	c	0.07 ± 0.01	c	62.01 ± 1.18	a	57.28 ± 0.59	b	0.05 ± 0.02	c	0.03 ± 0.00	c	61.10 ± 2.57	a	56.79 ± 0.81	b
Zn	R1	73.37 ± 3.05	a	74.63 ± 1.69	a	65.56 ± 1.94	b	65.37 ± 1.84	b	47.67 ± 1.77	c	47.80 ± 1.72	c	41.75 ± 1.03	d	40.87 ± 1.18	d
	R2	71.08 ± 4.45	a	66.17 ± 0.78	b	48.77 ± 2.14	c	43.90 ± 1.41	d	37.90 ± 1.04	e	40.27 ± 2.27	de	33.55 ± 1.54	f	33.47 ± 2.07	f
	R3	52.01 ± 1.71	a	48.22 ± 2.29	b	34.05 ± 0.82	c	31.29 ± 1.68	d	24.20 ± 1.66	e	23.93 ± 0.87	e	17.60 ± 1.55	f	16.47 ± 0.28	f
Mn	R1	118.52 ± 1.06	b	79.37 ± 1.47	c	52.20 ± 0.26	f	45.33 ± 2.88	f	133.03 ± 8.22	a	122.98 ± 6.39	b	70.68 ± 3.49	d	62.96 ± 4.57	e
	R2	181.20 ± 9.78	a	95.07 ± 3.84	c	64.91 ± 4.49	e	60.10 ± 6.05	e	121.38 ± 7.01	b	115.42 ± 7.24	b	90.21 ± 7.95	cd	83.33 ± 4.54	d
	R3	122.53 ± 9.39	a	93.55 ± 3.43	b	52.38 ± 5.78	d	52.03 ± 6.06	d	84.68 ± 3.30	b	74.60 ± 4.00	c	50.24 ± 4.40	d	51.72 ± 2.47	d
Cu	R1	25.13 ± 1.72	b	24.49 ± 0.88	b	14.17 ± 2.62	d	36.83 ± 1.14	a	19.19 ± 0.30	c	12.24 ± 1.10	d	12.89 ± 3.83	d	12.95 ± 0.96	d
	R2	16.98 ± 0.55	a	15.48 ± 1.34	b	17.86 ± 0.85	a	13.31 ± 1.30	c	12.48 ± 0.35	cd	11.20 ± 0.41	de	10.85 ± 0.30	e	10.40 ± 0.55	e
	R3	18.00 ± 0.76	a	14.32 ± 0.14	b	12.19 ± 0.36	c	10.81 ± 0.14	de	11.06 ± 0.36	d	7.51 ± 0.88	f	9.95 ± 0.85	e	9.89 ± 0.47	e
Fe	R1	96.49 ± 3.96	d	102.10 ± 7.09	cd	169.59 ± 8.86	a	162.76 ± 8.29	a	77.71 ± 1.77	e	97.06 ± 2.83	d	112.7 ± 9.10	bc	121.08 ± 10.54	b
	R2	94.15 ± 2.89	b	81.92 ± 4.16	c	114.01 ± 6.46	a	110.04 ± 1.41	a	75.35 ± 7.13	c	72.21 ± 5.45	c	119.32 ± 7.25	a	117.29 ± 10.22	a
	R3	77.49 ± 3.92	bc	72.34 ± 3.08	cd	106.11 ± 4.93	a	84.46 ± 8.94	b	61.62 ± 3.97	e	64.90 ± 2.69	de	110.39 ± 9.31	a	62.37 ± 4.96	e

The data were the means of three independent replicates. The different letters in each line indicate the significant differences (*p* < 0.05) among the 4 treatments within two cultivars. S1, S2, and S3 correspond to the top part of the shoot (three top leaves with stem), the middle part of the shoot, and the bottom part of the shoot (three bottom leaves with stem), respectively; R1, R2, and R3 correspond to the top part of the root (three cm of root top), the middle part of the root, and the bottom part of the root (three cm of root bottom), respectively. Control, GB, Cd, and Cd + GB correspond to BNS + foliar spray of deionized water, BNS + foliar spray of 500 μM GB, BNS + foliar spray of deionized water + 5 μM CdCl_2_, and BNS + foliar spray of 500 μM GB + 5 μM CdCl_2_, respectively.

## Supplementary Materials

Supplementary materials can be found at https://www.mdpi.com/1422-0067/20/7/1612/s1. **Figure S1.** The effects of Cd and exogenous GB application on MDA accumulation in the shoots (**A**,**B**) and roots (**C**,**D**) of two tobacco cultivars (Left: Guiyan1; Right: Yunyan2) exposed to 5 µM Cd for 5, 10, and 15 days: The error bars represent the SD values (*n* = 3). The different letters indicate the significant differences (*p* < 0.05) among the 4 treatments within each sampling date. Control, GB, Cd, and Cd + GB correspond to BNS + foliar spray of deionized water, BNS + foliar spray of 500 μM GB, BNS + foliar spray of deionized water + 5 μM CdCl_2_, and BNS + foliar spray of 500 μM GB + 5 μM CdCl_2_, respectively. **Figure S2.** The effects of Cd and exogenous GB application on the SOD, POD, CAT, and APX activities in the shoots of two tobacco cultivars (Left: Guiyan1; Right: Yunyan2) exposed to 5 µM Cd for 5, 10, and 15 days: The error bars represent the SD values (*n* = 3). The different letters indicate the significant differences (*p* < 0.05) among the 4 treatments within each sampling date. Control, GB, Cd, and Cd + GB correspond to BNS + foliar spray of deionized water, BNS + foliar spray of 500 μM GB, BNS + foliar spray of deionized water + 5 μM CdCl_2_, and BNS + foliar spray of 500 μM GB + 5 μM CdCl_2_, respectively. **Figure S3.** The effects of Cd and exogenous GB application on the photosynthesis parameters of two tobacco cultivars after 15 days of Cd exposure: The error bars represent the SD values (*n* = 3). The different letters indicate the significant differences (*p* < 0.05) among the 4 treatments within each cultivar. Control, GB, Cd, and Cd + GB correspond to BNS + foliar spray of deionized water, BNS + foliar spray of 500 μM GB, BNS + foliar spray of deionized water + 5 μM CdCl_2_, and BNS + foliar spray of 500 μM GB + 5 μM CdCl_2_, respectively. Pn = net photosynthetic rate, gs = stomatal conductance, Tr = transpiration rate, and Ci = intercellular CO_2_ concentration. **Figure S4.** Transmission electron micrograph images of the root tip cells of Guiyan1 (left panel) and Yunyan2 (right panel) after 15 days of Control (**A**,**B**), GB (**C**,**D**), Cd (**E**,**F**), and Cd + GB (**G**,**H**) treatments, respectively. Control, GB, Cd, and Cd + GB correspond to BNS + foliar spray of deionized water, BNS + foliar spray of 500 μM GB, BNS + foliar spray of deionized water + 5 μM CdCl_2_, and BNS + foliar spray of 500 μM GB + 5 μM CdCl_2_, respectively. Labels: N, nuclear; NL, nucleolus; V, vacuole; W, cell wall; M, mitochondrion, PL, plasmolysis. The arrows indicate the electron dense granules (EDG). Bar = 1 μm. **Figure S5.** The effects of Cd and GB on the stomatal aperture of Guiyan1 (left panel) and Yunyan2 (right panel) after 15 days of Control (**A**,**B**), GB (**C**,**D**), Cd (**E**,**F**), and Cd + GB (**G**,**H**) treatments, respectively. Control, GB, Cd, and Cd + GB correspond to BNS + foliar spray of deionized water, BNS + foliar spray of 500 μM GB, BNS + foliar spray of deionized water + 5 μM CdCl_2_, and BNS + foliar spray of 500 μM GB + 5 μM CdCl_2_, respectively. The photos were detected by a scanning electron microscope. Labels: OS, open stomata with aperture width ≥ 2 μm; CS, close stomata with aperture width < 2 μm; EH, epidermal hair. Bar = 20 μm.
