# Supplementary material for: Exogenous Glycinebetaine Reduces Cadmium Uptake and Mitigates Cadmium Toxicity in Two Tobacco Genotypes Differing in Cadmium Tolerance
Source: Int J Mol Sci. 2019 Mar 31;20(7):1612. doi: 10.3390/ijms20071612 (PMC6480294; doi:10.3390/ijms20071612)
Supplement: Supplementary file 1 [file ijms-20-01612-s001.pdf]

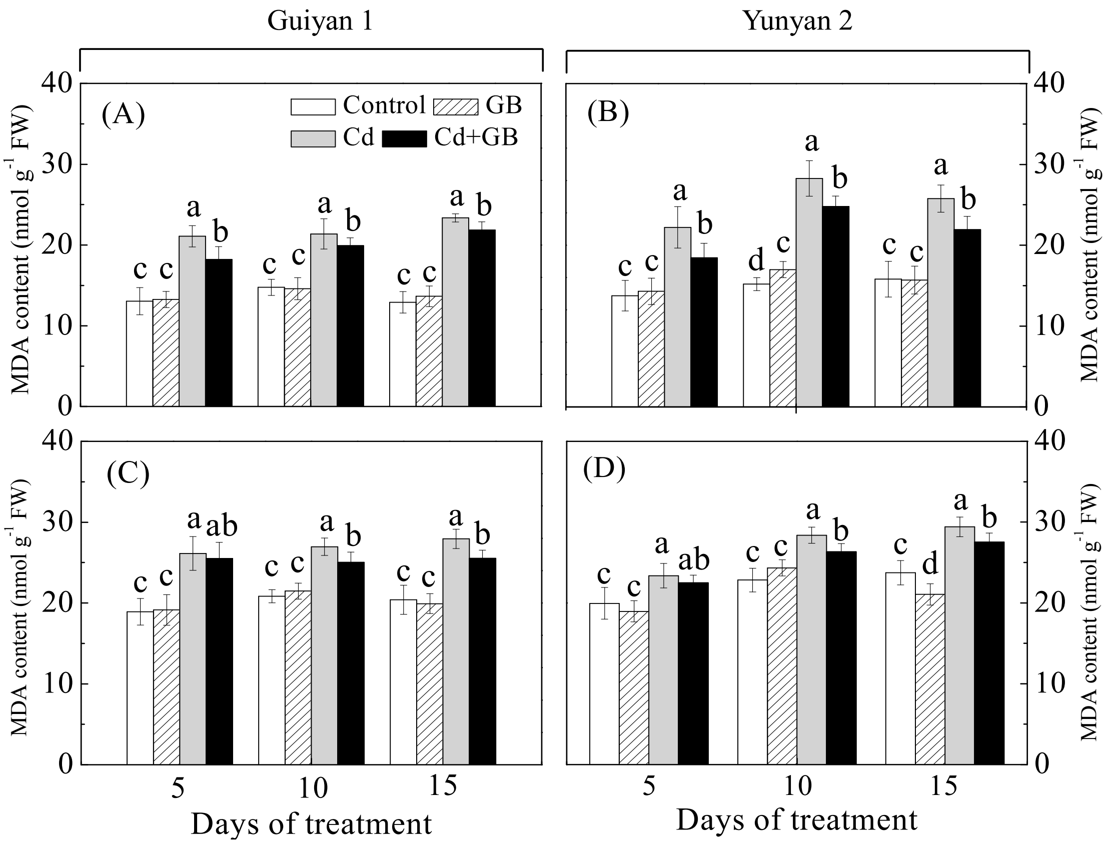

Figure S1. The effects of Cd and exogenous GB application on MDA accumulation in the shoots (**A**,**B**) and roots (**C**,**D**) of two tobacco cultivars (Left: Guiyan1; Right: Yunyan2) exposed to 5 μM Cd for 5, 10, and 15 days: The error bars represent the SD values (n = 3). The different letters indicate the significant differences ($p < 0.05$) among the 4 treatments within each sampling date. Control, GB, Cd, and Cd + GB correspond to BNS + foliar spray of deionized water, BNS + foliar spray of 500 μM GB, BNS + foliar spray of deionized water + 5 μM $CdCl_2$, and BNS + foliar spray of 500 μM GB + 5 μM $CdCl_2$, respectively.

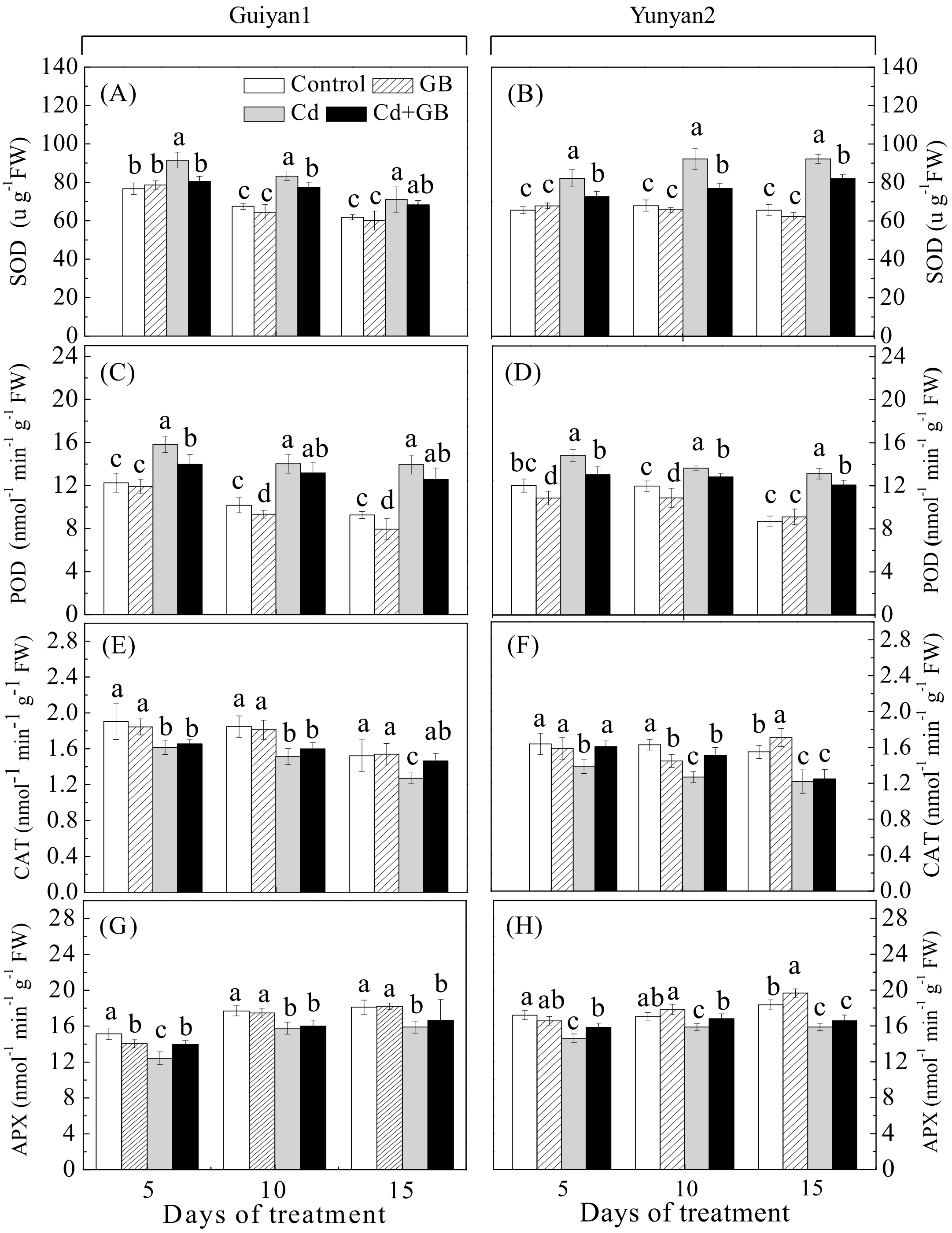

Figure S2. The effects of Cd and exogenous GB application on the SOD, POD, CAT, and APX activities in the shoots of two tobacco cultivars (Left: Guiyan1; Right: Yunyan2) exposed to 5 μM Cd for 5, 10, and 15 days: The error bars represent the SD values (n = 3). The different letters indicate the significant differences ($p < 0.05$) among the 4 treatments within each sampling date. Control, GB, Cd, and Cd + GB correspond to BNS + foliar spray of deionized water, BNS + foliar spray of 500 μM GB, BNS + foliar spray of deionized water + 5 μM $CdCl_2$, and BNS + foliar spray of 500 μM GB + 5 μM $CdCl_2$, respectively.

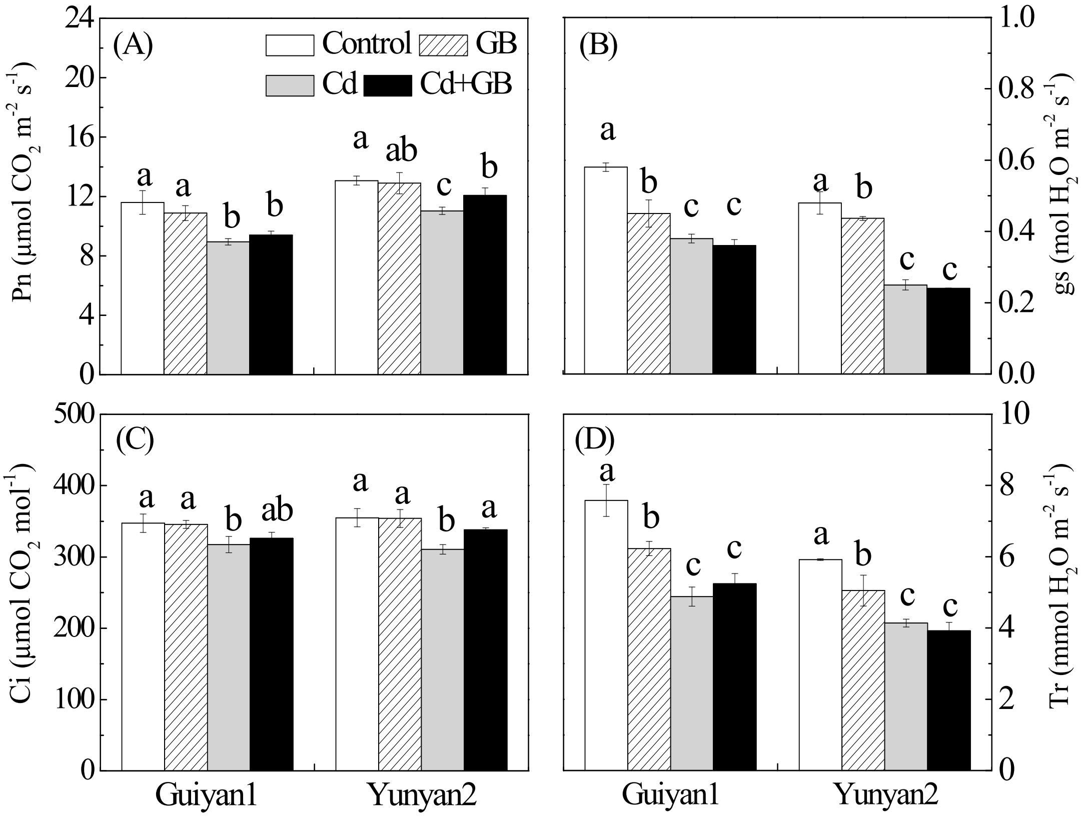

Figure S3. The effects of Cd and exogenous GB application on the photosynthesis parameters of two tobacco cultivars after 15 days of Cd exposure: The error bars represent the SD values (n = 3). The different letters indicate the significant differences (p < 0.05) among the 4 treatments within each cultivar. Control, GB, Cd, and Cd + GB correspond to BNS + foliar spray of deionized water, BNS + foliar spray of 500 μM GB, BNS + foliar spray of deionized water + 5 μM $CdCl_2$, and BNS + foliar spray of 500 μM GB + 5 μM $CdCl_2$, respectively. Pn = net photosynthetic rate, gs = stomatal conductance, Tr = transpiration rate, and Ci = intercellular CO_2 concentration.

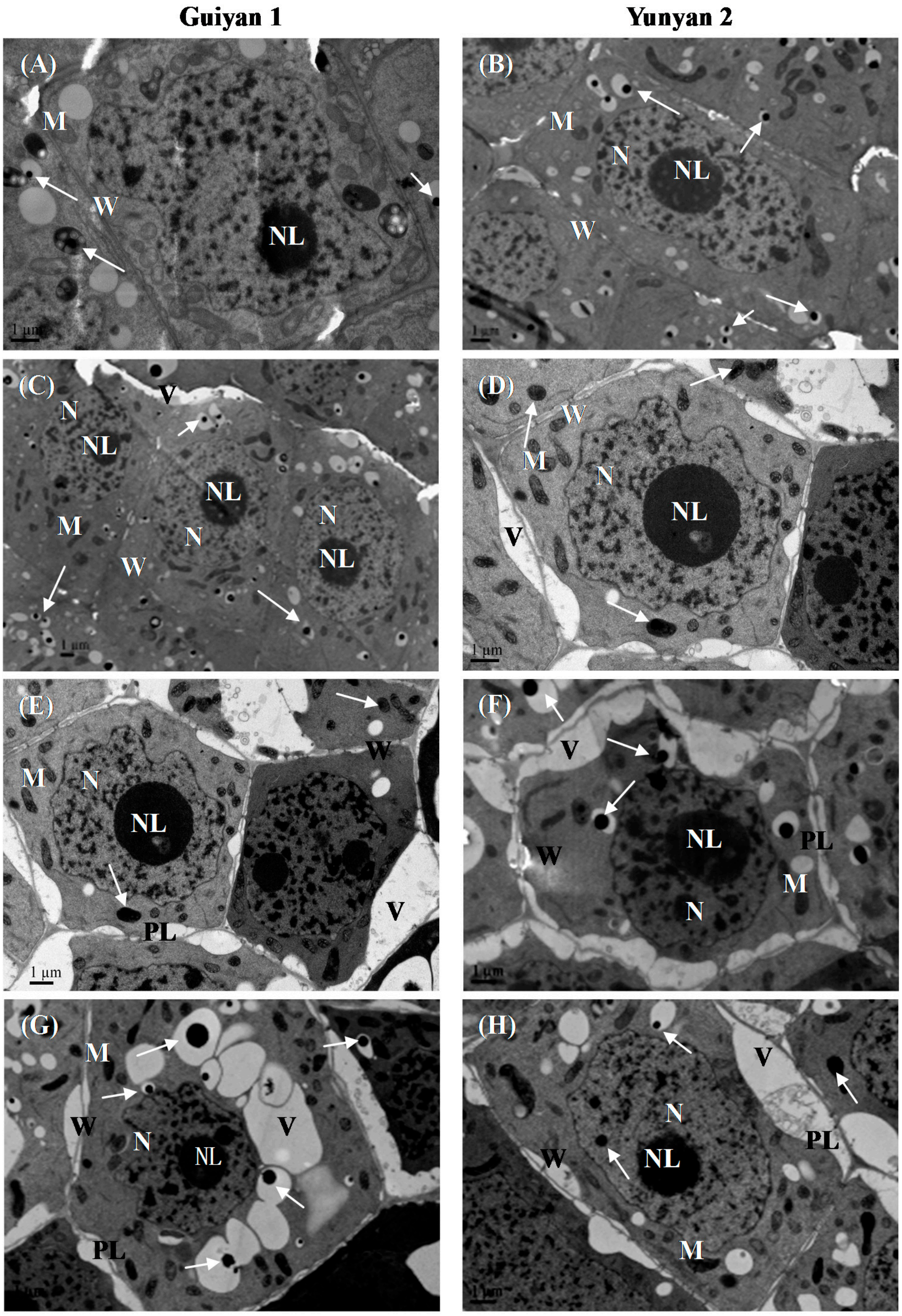

Figure S4. Transmission electron micrograph images of the root tip cells of Guiyan1 (left panel) and Yunyan2 (right panel) after 15 days of Control (**A**,**B**), GB (**C**,**D**), Cd (**E**,**F**), and Cd + GB (**G**,**H**) treatments, respectively. Control, GB, Cd, and Cd + GB correspond to BNS + foliar spray of deionized water, BNS + foliar spray of 500 μM GB, BNS + foliar spray of deionized water + 5 μM $CdCl_2$, and BNS + foliar spray of 500 μM GB + 5 μM $CdCl_2$, respectively. Labels: N, nuclear; NL, nucleolus; V, vacuole; W, cell wall; M, mitochondrion, PL, plasmolysis. The arrows indicate the electron dense granules (EDG). Bar = 1 μm.

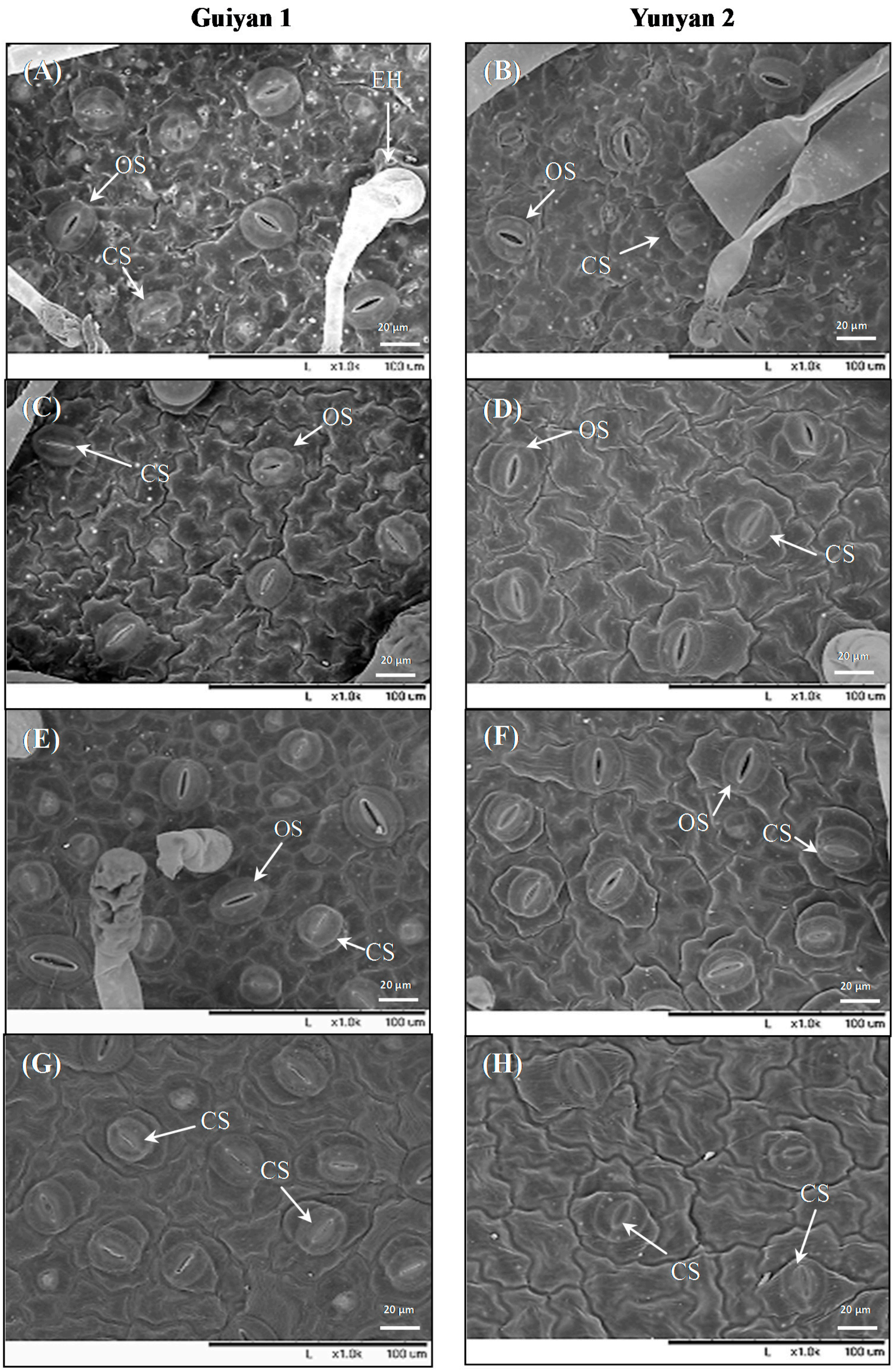

Figure S5. The effects of Cd and GB on the stomatal aperture of Guiyan1 (left panel) and Yunyan2 (right panel) after 15 days of Control (**A**,**B**), GB (**C**,**D**), Cd (**E**,**F**), and Cd + GB (**G**,**H**) treatments, respectively. Control, GB, Cd, and Cd + GB correspond to BNS + foliar spray of deionized water, BNS + foliar spray of 500 μM GB, BNS + foliar spray of deionized water + 5 μM $CdCl_2$, and BNS + foliar spray of 500 μM GB + 5 μM $CdCl_2$, respectively. The photos were detected by a scanning electron microscope. Labels: OS, open stomata with aperture width ≥ 2 μm; CS, close stomata with aperture width < 2 μm; EH, epidermal hair. Bar = 20 μm.
